# Comparison of the multi-drug resistant human hepatocellular carcinoma cell line Bel-7402/ADM model established by three methods

**DOI:** 10.1186/1756-9966-29-115

**Published:** 2010-08-20

**Authors:** Xingguo Zhong, Maoming Xiong, Xiangling Meng, Renhua Gong

**Affiliations:** 1 Department of General Surgery, The First Affiliated Hospital of Anhui Medical University, Hefei 230022, China; 2 Department of General Surgery, The Anhui Provincial Corps Hospital of Chinese People's Armed Police Force, Hefei 230041, China

## Abstract

**Background:**

To compare the biological characteristics of three types of human hepatocellular carcinoma multi-drug resistant cell sub-lines Bel-7402/ADM models established by three methods.

**Methods:**

Established human hepatocellular carcinoma adriamycin (ADM) multi-drug resistant cell sub-lines models Bel-7402/ADM_V_, Bel-7402/ADM_L _and Bel-7402/ADM_S _by three methods of in vitro concentration gradient increased induction, nude mice liver-implanted induction and subcutaneous-implanted induction respectively. Phase contrast microscopy was used to observe the cells and the MTT (methyl thiazolyl tetrazolium) method was used to detect drug resistance of the three different sub-lines of cells.

**Results:**

The three groups of drug resistant cells, Bel-7402/ADM_V_, Bel-7402/ADM_L _and Bel-7402/ADM_S _generated cross-resistance to ADM and CDDP (cis-Diaminedichloroplatinum), but showed a significant difference in resistance to Bel-7402 IC_50 _value (*P *< 0.01). The doubling times were significantly extended compared to the parent cell line (39 h) and were 65 h (Bel-7402/ADM_V_), 46 h (Bel-7402/ADM_L_), and 45 h (Bel-7402/ADM_S_). The excretion rates of ADM were significantly increased compared with the parent cell (34.14%) line and were 81.06% (Bel-7402/ADM_V_), 66.56% (Bel-7402/ADM_L_) and 61.56% (Bel-7402/ADM_S_). Expression of P-gp and MRP in the three groups of resistant cells was significantly enhanced (*P *< 0.01). There was no significant variation in the expression of GSH/GST (*P *> 0.05).

**Conclusions:**

Stable resistance was involved in the resistant cell line model established by the above three methods. Liver implantation was a good simulation of human hepatocellular and proved to be an ideal model with characteristics similar to human hepatocellular biology and the pharmacokinetics of anticancer drugs.

## Background

The current treatment of hepatocellular carcinoma, especially hepatocellular carcinoma in middle and advanced stages, is a comprehensive therapy using a combination of surgery and chemotherapy. Chemotherapy plays a critical role in the treatment of hepatocellular carcinoma. Nevertheless, multi-drug resistance (MDR) [1,2] of hepatocellular carcinoma cells to multiple chemotherapeutics renders chemotherapy for hepatoma insufficient. Therefore, the target of drug resistance and its reverse strategy is one of the hotspots of hepatocellular carcinoma research.

Establishing a reliable tumor MDR model is the foundation for the study of tumor MDR and its reversal. In this study, we established three different human hepatocellular carcinoma drug-resistance cell sub-lines of Bel-7402/ADM by applying ADM by three normal methods. We compared the biological characteristics the three cell sub-lines to acquire a comparatively ideal drug-resistance model which paved the way for revealing the clinical multidrug resistance phenomenon and the screening of a reversal agent.

## Materials and methods

### Cells and Animals

Human hepatocellular carcinoma cell line Bel-7402 was purchased from Shanghai Institute of Biological Products. Four-week-old BALB/c-nu/nu nude mice weighting 12-16 g were purchased from Shanghai Shilaike Co., Ltd., and were bred in the specific pathogen free (SPF)Animal Center, School of Life Science, University of Science and Technology of China.

### Establishment of a multi-drug resistance cell model based on nude mice liver implantation and subcutaneous implantation

A total of 20 male nude mice aged 4-6 weeks were used. Ten mice were anesthesized by an intraperitoneal injection with chloral hydrate (430 mg/kg). A transverse incision was performed under the xiphoid process. A 0.2-ml Bel-7402 cell suspension (density equal to 1 × 10^8^/ml) was injected into the parenchyma of the right hepatic lobe and the abdomen was closed. The ten mice were randomly divided into the liver implantation experimental group or the control group with equal members (n = 5 for each group). Another 10 animals were subcutaneously injected with 0.2-ml Bel-7402 cell suspension (density equal to 1 × 10^8^/ml) into the right anterior axilla. they were also randomly divided into experimental and control groups (n = 5 for each group). All animals were bred in SPF condition. On the third day, nude mice in the experimental groups underwent an intraperitoneal injection with ADM at a dose of 1.5 mg/kg each week for 8 weeks. Mice in the control groups underwent an intraperitoneal injection with an equal volume of normal saline solution. Skin reaction, appetite and psychological status were recorded according to the observation in each day. The tumor volume was calculated by the following formula: V = πab^2^/a ("a" represents the long diameter of the tumor, "b" represents the short diameter of the tumor). When the experiment was completed, the nude mice were sacrificed, the tumor was obtained and levigated in asepsis. A 0.25% trypsin solution was used to digest the cells for 2-3 min and to produce a mono-cell suspension. Cells were inoculated in a 25-ml sterile culture flask for primary culture. After multiple passages and purification, the hepatocellular implantation drug-resistant cell sub-lines Bel-7402/ADM_L _(liver-implanted induction) and the subcutaneous implantation drug-resistant cell sub-lines Bel-7402/ADM_S _(subcutaneous-implanted induction) were obtained. Tumor tissue was fixed with 1% osmium tetroxide, embedded in resin, and cut into ultra thin sections. After uranyl acetate and citric acid double staining, the sections were observed by an transmission electron microscope (Zeiss 902).

### Establishment of a multi-drug resistance model by in vitro induction

The ADM concentration gradient progressive increase induction method was applied. Bel-7402 cells at a concentration of 5 × 10^5^/ml in the logarithmic phase were inoculated in a 25-ml culture flask and cultured for 24 h. The culture solution was replaced with an ADM culture solution at a low concentration (0.01 μg/ml). After the 24-h culture, the solution containing drugs was discarded. Cells were digested with 0.25% trypsin and centrifuged at 1000 rpm for 3 min. The cells were collected and re-inoculated in a 25-ml culture flask containing a solution without ADM at a concentration of 1 × 10^5^/ml. While cell growth was in the logarithmic phase, drug concentration was elevated and the extent of improvement increased the cell survival rate 60-70%. This protocol was repeated for a period of approximately 6 months, until the cells exhibited stable growth and proliferation in a culture medium with 0.5 μg/ml ADM. This cell sub-lines named Bel-7402/ADM_V _(vitro induction).

### Detection of cellular sensitivity to drug by MTT (methyl thiazolyl tetrazolium) methods

Four groups of cells (the parent cell line and the three different groups of drug-resistant cell sub-lines) in the logarithmic phase of growth were obtained for the preparation of cell suspension. Cell concentration was adjusted to 5 × 10^5^/ml and 200 μl (approximately 10^5 ^cells) was placed in each well of a 96-well culture plate. After a 24-h culture, the following investigational drugs were added: ADM, CDDP, MMC, MTX and 5-FU. In accordance with peak blood concentrations of a clinical dose of each drug, the concentration range was varied from 10^3^- to 10^-3^-fold of peak blood concentrations. Seven diverse experimental concentrations were defined as follows: 10^3^, 10^2^, 10^1^, 10^0^, 10^-1^, 10^-2 ^and 10^-3 ^fold of peak blood concentration. A control group without drugs was also set and included five different duplicate wells in each experimental concentration. All cells were cultured at 37°C and 5% CO_2 _for 24 h. Twenty microliters of an MTT (5 mg/ml) solution was added to each well and cells were cultured for an additional 4 h. Supernatants were discarded after termination of the culture and 150 μl of dimethyl sulphoxide (DMSO) was added to each well. Plates were shaken for 10 min and a microplate reader was used to measure the optical density (OD) value at a wavelength of 570 nm (the correction wavelength was 630 nm) to calculate cell survival rate. The following equation was used to calculate cell survival rate: cell survival rate = (the OD value in each experiment well/the OD value in the control well) ×100%. The 50% of inhibition concentration (IC_50_) of drug was measured by chartography. The resistance index (RI) = the IC_50 _of drug-resistant cells/the IC_50 _of parent cell line. MTT experiments were repeated three times on different days.

### Plotting of the growth curve and measurement of doubling time

Four groups of cells with an excellent growth condition were obtained and RPMI- 1640 complete culture solution was applied to prepare a cell suspension (5 × 10^3^/ml) of each. A 6-well plate (1 ml/well) was inoculated. Cell counting was performed after 1, 2, 3, 4, 5, 6, or 7 d of inoculation, when 3 pores were obtained for each day and mean values were obtained. The culture time was set as the X-axis and cell numbers were set as the Y-axis to draw the growth curve. According to the Patterson equation, cell doubling time was calculated as follows: Td = tlg2/lg (Nt/N0) where Td: doubling time (h); t: required time when cell numbers increased from N0 to Nt; N0: cell numbers in the inoculation; Nt: cell numbers after culture for t hours.

### Uptake and excretion of ADM

Flow cytometry was used to measure fluorescence intensity of ADM and to reflect its concentration indirectly. Four groups of cells in the logarithmic phase of growth were obtained to prepare a cell suspension of 1 × 10^6^/ml cells. ADM was added to a final concentration of 4.0 μg/ml. Cells were placed in a CO_2 _incubator for 20 min, and then a 1-ml solution was obtained for centrifugation. Cold PBS was used to wash the cells twice and they were resuspended in 0.5 ml PBS. The relative fluorescent intensity of ADM was detected by flow cytometry immediately (excitation wavelength was 479 nm, emission wavelength was 587 nm). In the excretion experiment, the above cells were centrifuged, washed in cold RPMI-1640 culture solution, re-suspended in culture solution without adding drug and placed in a CO_2 _incubator for 60 min. After this incubation period, cells were centrifuged, washed with PBS and the relative fluorescence intensity of ADM was detected by flow cytometry. The excretion rate of ADM reflected the excretive function of ADM by cells. The excretion rate of ADM = 100% × (uptake value - stagnation value)/uptake value. Experiments were repeated 5 times at different time points.

### Measurements of P-glycoprotein (P-gp), multidrug resistance-associated protein (MRP) and the expression of glutathione S-transfer enzyme system (GSH/GST) detected by flow cytometry

The four groups of drug-resistant cells and parent cells in the logarithmic phase of growth (1 × 10^8^/ml) were obtained with five tubes in each group. PBS (4°C, 0.01 mol/l, pH 7.4) was applied twice then MRK16(MDR1), MRPrl (MRP) and GSH/GST mouse-anti-human monoclonal antibody were added for 1 h at 4°C. The mouse-anti-human isotype-matched monoclonal antibody was applied as a control Goat-anti-mouse fluorescent labeled IgG was added, incubated at 4°C for 30 min, and fluorescence intensity was detected by flow cytometry.

### Statistical analysis

All data are expressed as the mean ± SD and analyses were carried out using SPSS10.0 software (SPSS Inc, Chicago, IL). The Student's t-test and one-way ANOVA were used for comparisons among the means. A p-value less than 0.05 was considered statistically significant.

## Results

### Drug-resistant model of subcutaneous and liver implantation tumors

**Figure 1:**
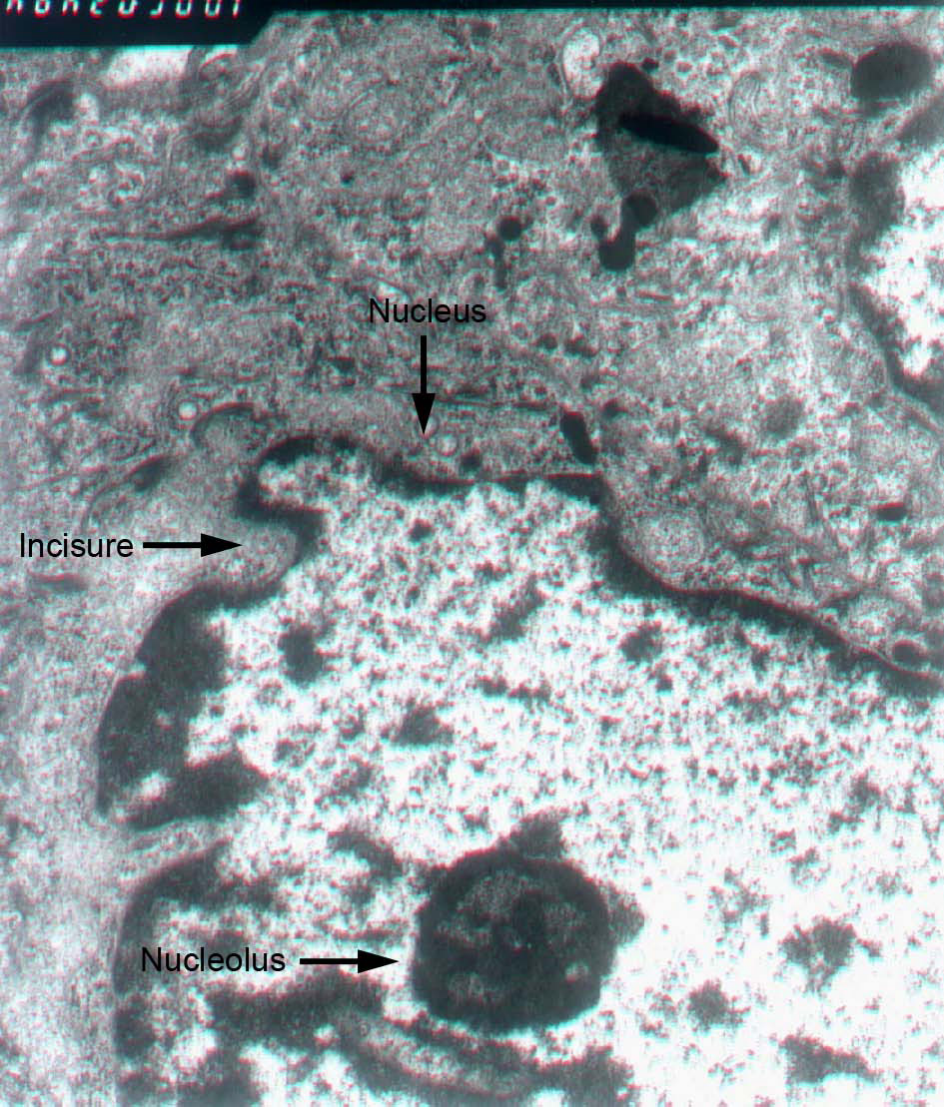
**Under electron microscope, the tumor cells share many similarities with human hepatocellular carcinoma cells, including enlarged nuclei, hyperchromatic nucleoli, and multiple nuclear membrane incisures**. (×10000).

The subcutaneous implanted tumors were all successfully inoculated (10/10). The mean incubation periods in the experimental group and the control group were 18 ± 6 d. The growth of tumors in the experimental group was 3.60 ± 0.58 mm^3^/day, whereas in the control group, it was 3.75 ± 0.26 mm^3^/day. The 10 nude mice with liver implanted tumors were all successfully inoculated. The growth of tumors in the experimental group was 3.50 ± 0.37 mm^3^/day, whereas in the control group, it was 3.70 ± 0.41 mm^3^/day. During the 8-wks breeding and induction of ADM, tumors in each group grew well, with ruddy skin. In the liver implanted group, 2 mice developed abdominal dropsy, but no cachexia or death occurred. After 8 wks, all nude mice were sacrificed. The general morphology of the implanted tumor in both the experimental and control groups showed no significant difference. Tumors assumed an ellipse or irregular sublobe morphology. Under electron microscope, the tumor cells share many similarities with human hepatocellular carcinoma cells, including enlarged nuclei, hyperchromatic nucleoli, and multiple nuclear membrane incisures (Figure 1). The mean tumor weight was 1.48 ± 0.21 g. Fibrous tissue abundantly surrounded the tumor. The incisal surface of the tumor body was gray. The minority of tumors showed a scattered and clustered distribution. In addition, three mice exhibited metastases in the abdominal cavity in the liver implanted group.

### Observation of cell morphology

Under a phase contrast microscope, Bel-7402 cells were fusiform, aligned and compact with well-distributed sizes, distinct boundaries and growth with adherence. After the addition of drugs, the majority of cells appeared apoptotic and subsequently dissolved. The size of the surviving cells was unequal, the cellular profile was unclear and adherence was reduced. After about two weeks, cell growth recovered and the above variations in the acute stage had disappeared. The morphology of resistant-cells was irregular, with slightly augmented volume, which signifies accumulated growth. Massive particles and vacuoles appeared in the cytoplasm and the nucleus exhibited slight shrinkage.

### Sensitivity of the three types of cell sub-lines toward anticancer drugs (Table 1)

Table 1 indicates that the three resistant cell sub-lines generated cross-resistance toward ADM and CDDP but showed no cross-resistance to mitomycin (MMC), methotrexate (MTX), 5 -fluorouracid (5- FU).

**Table 1:** Sensitivity of Bel-7402/ADM_S_, Bel-7402/ADM_L _and Bel-7402/ADM_V _cells to multiple chemotherapy drugs.

Drug	**IC**_**50 **_**(mg.L**^**-1**^, $\bar{x}$** ± *s*)**				RI		
		
	Bel-7402	**Bel-7402/ADM**_**S**_	**Bel-7402/ADM**_**L**_	**Bel-7402/ADM**_**V**_	**RI**_**S**_	**RI**_**L**_	**RI**_**V**_
ADM	2.09 ± 0.13	26.69 ± 0.46	26.92 ± 0.38	46.93 ± 0.82	12.77	12.88	22.45
CDDP	0.98 ± 0.11	12.92 ± 3.45	13.46 ± 3.00	25.18 ± 3.57	13.18	13.73	25.69
MMC	0.54 ± 0.05	0.57 ± 0.08	0.60 ± 0.08	0.62 ± 0.04	1.06	1.11	1.15
MTX	0.15 ± 0.05	0.17 ± 0.05	0.20 ± 0.06	0.21 ± 0.05	1.13	1.33	1.4
5-FU	119.65 ± 6.46	120.78 ± 4.84	121.60 ± 6.15	123.66 ± 5.00	1.01	1.02	1.03

Note: By least significant difference (LSD) paired-comparison in both ADM and CDDP groups, except Bel-7402/ADM_L _vs. Bel-7402/ADM_S _(P > 0.05), there is no statistical significance. In other groups of resistant cells, there is a significant difference by paired-comparison. In addition, in MMC, MTX and 5-FU groups, there is no statistical significance by paired-comparison (P > 0.05).

### Growth curve and doubling time (Figure 2)

The doubling time of drug-resistant cells was significantly extended compared with parent cells. The doubling times in Bel-7402, Bel-7402/ADM_S_, Bel-7402/ADM_L _and Bel-7402/ADM_V _cells were 39 h, 45 h, 46 h and 65 h, respectively.

**Figure 2:**
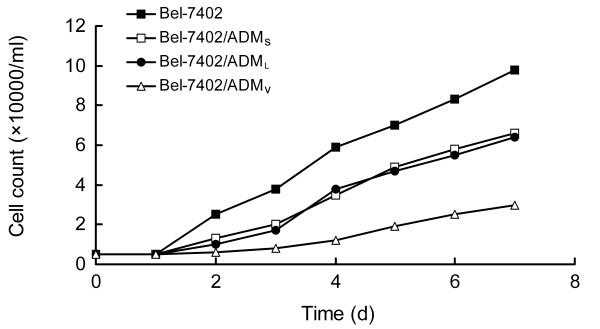
**Cells growth curve**. The doubling time of the cells was proportional to the drug-resistance of cell lines.

### Uptake and excretion of ADM (Table 2)

The excretion rate of Bel-7402, Bel-7402/ADM_S_, Bel-7402/ADM_L _and Bel-7402/ADM_V _cells to ADM were 34.14%, 61.56%, 66.56% and 81.06%, respectively. The relative fluorescent intensity in each group of cells was reduced after the excretion of ADM and drug-resistant cells were more obvious compared with parent cells.

**Table 2:** Cellular relative fluorescent intensity after the uptake and excretion of ADM.

Cell	Cellular relative fluorescence intensity of ADM		Excretion rate of ADM (%)
	After Uptake	After Excretion	
Bel-7402 (Parent)	11.19 ± 0.23	7.37 ± 0.16	34.14
Bel-7402/ADM_S_	15.27 ± 0.22	5.87 ± 0.13	61.56
Bel-7402/ADM_L_	15.61 ± 0.18	5.22 ± 0.13	66.56
Bel-7402/ADM_V_	19.11 ± 0.15	3.62 ± 0.17	81.06
F	1338.016	531.312	
P	0.000	0.000	

Note: By LSD paired-comparison after the uptake and excretion, drug-resistant cellular relative fluorescent intensity of ADM showed significant differences (*P *< 0.05).

### Variation of expression of P-gp, MRP and GSH/GST detected by flow cytometry (Table 3)

**Figure 3:**
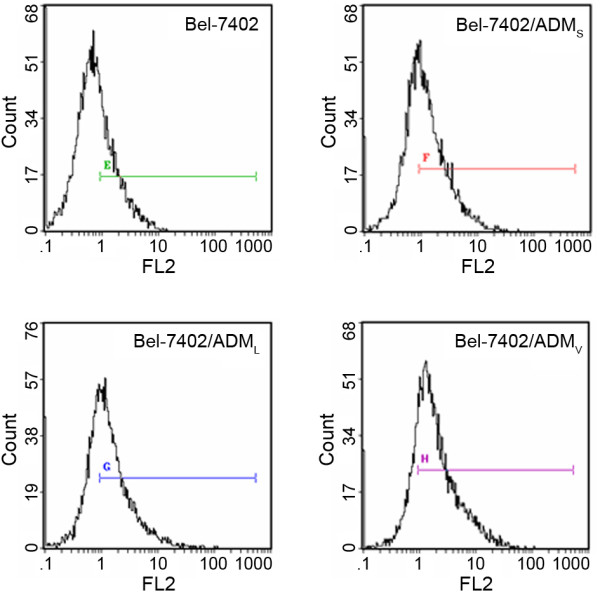
**The flow cytometry histograms of MRP expression**. With the MRP fluorescence staining rate increased gradually in the four groups, the peak dramatically moves to the right of the coordinate system.

Expression of P-gp in the three groups of the resistant cells was significantly enhanced (*P *< 0.01). The MRP fluorescence staining rates were also significantly raised in the three groups of drug resistant cells, the in vitro induction group with the highest rate, the other two groups relatively lower. It is shown that the peak dramatically moves to the right of the coordinate system (Figure 3). The expression of GSH/GST in the three groups showed no statistical significance by paired-comparison (*P *>0.05).

**Table 3:** Staining rate of P-gp, MRP and GSH/GST fluorescent cells analyzed by flow cytometry.

Cell	**Expression rate (%, **$\bar{x}$** ± *s*)**		
	
	P-gp	MRP	GSH/GST
Bel-7402 (Parent)	19.59 ± 0.62	21.29 ± 1.14	26.92 ± 1.79
Bel-7402/ADM_S_	65.92 ± 1.41	56.88 ± 1.49	27.76 ± 1.00
Bel-7402/ADM_L_	68.10 ± 1.88	58.84 ± 2.35	28.97 ± 1.42
Bel-7402/ADM_V_	91.93 ± 2.49	78.28 ± 1.23	27.57 ± 1.24
*F*	1512.300	1064.757	1.890
*P*	0.000	0.000	0.172

Notes: By LSD paired-comparison in both P-gp and MRP groups, except for Bel-7402/ADM_L _vs. Bel-7402/ADM_S _(P > 0.05), there was no statistical significance. In other groups of resistant cells, there was a significant difference by paired-comparison (P < 0.01). In addition, for GSH/GST, there was no statistical significance by paired-comparison (P > 0.05).

## Discussion

### Current condition and progression of hepatoma therapeutics

Hepatic cell carcinoma is generally accepted as one of the most harmful malignant tumors, with high malignancy and a bad prognosis. Recent years have witnessed an uprising in the incidence rate of hepatoma. Therefore, it is of vital importance to improve the therapeutic treatment of hepatoma. Excision is still the best alternative in the multiple therapeutic methods for the treatment of hepatoma [3,4]. Nevertheless, the diagnostic rate in earlier hepatoma is quite low and the progression of disease is comparatively rapid. Therefore, the majority of patients have lost a surgical opportunity after final diagnosis. References indicate that 60% of patients have clinical or endoscopic metastasis in the final diagnosis of hepatoma [5]. Thus, non-operative therapy showed better practical value than operative therapy. Chemotherapy is also commonly used in non-operative methods, and is a kind of general therapeutic method for the treatment of the primary tumors, metastases and inferior clinical metastatic tumors. However, the involvement of MDR seriously affects the chemotherapeutic effect in hepatoma.

### Significance of the establishment of multi-drug resistant human hepatocellular carcinoma cell sub-lines model

The chemotherapeutic effect was restricted due to the involvement of multi-drug resistance of hepatocellular carcinoma cells. The related MDR of hepatoma and its clinical reversal is becoming a critical clinical problem that needs a further solution. Research on this aspect requires the establishment of a reliable multi-drug resistant cell model [6].

Currently, the establishment of a multi-drug resistant human hepatocellular carcinoma cell line model includes methods such as the application of an in vitro culture to induce tumor MDR, multi-drug resistant gene transfection and the induction of drug-resistance by nude mice implanted model. Induction of tumor MDR in vitro culture also required two types of methods, the drug concentration incremental gradient method and the high-concentration intermittent drug-induced method [7,8]. The drug-resistance method induced by nude mouse in vivo transplantation includes three methods: subcutaneous implantation, liver implantation and abdominal implantation. There are advantages and disadvantaged involved in the various methods. In vitro drug concentration incremental gradient induction, liver and subcutaneous implanted induction of nude mice are commonly used as three methods for establishing multi-drug resistant human ADM hepatocellular carcinoma cell sub-lines. The tumor cell microenvironment includes various factors such as temperatures, pH values, local oxygen concentration, cell matrix, nutritional condition and medications, which play a critical regulatory role in the biological behavior of cells and MDR expression. Therefore, further clarification on methods that can establish drug-resistant cell models that accurately reflect the practical process of clinical drug-resistance are needed. Our experiment aimed to identify which drug-resistant cell line is the ideal model for the study of the mechanism of hepatoma drug-resistance and paves a way for the further investigation of drug-resistant and its reversal.

### Comparisons of three drug-resistance models

The induction of multi-drug resistance in tumor cells was caused by factors such as P-gp [9], MRP, LRP, GST, glutathione, glutathione S-transferase, protein kinase C, apoptosis-related gene (bcl-2, c-myc, p53), and the high-expression of GCS in the cancer cell living environment and variation of DNA type II topoisomerase activity [10-17]. As the drug-resistant mechanism of tumors is quite complicated, the drug-resistant phenotype of MDR cells was contained in cell specificity, distinct inductive medicines and diverse induction methods, the concluded drug-resistant phenotype was not quite uniform [18-20]. In our experiment, we compared three types of multi-drug resistant human hepatocellular carcinoma cell sub-lines ADM model established by three methods. The summary is shown below.

### Comparisons of biological characteristics in the three models

The morphology of each drug-resistant cell line was irregular, volume was slightly increased compared with the parental generation, growth velocity was slower which enables accumulative growth, cell boundaries were obscure, massive particles and vacuoles were observed in the cytoplasm, and a slight shrinkage of the nucleus appeared. The in vitro induction of drug-resistant cells showed significant differences and the morphology of drug-resistant cell induced by in vivo implantation was close to the parental generation.

The doubling times of the three drug-resistant cell lines, which were significantly extended compared with the parent cell line, revealed that growth velocity and the reproductive activity of the drug-resistant cell line applied by an in vitro concentration gradient incremental method was significantly lower than that of the other two kinds of in vivo inductions.

For the mechanisms of drug-resistance, the higher increase of drug excretion induced by a drug efflux pump was one of the most common drug-resistant reactions [21]. For this reason, we detected and compared the influx and efflux of ADM in three kinds of cells. The results indicated that the efflux rates of the four groups were 34.14%, 61.56%, 66.56% and 81.06%. Efflux rate of ADM by the resistant cell was significantly increased which was reflected as the drug stagnation diminished. This caused the intracellular drug concentration to decrease and diminish the impairment of cell target organs by drugs, which is presumed to be the main cause of the higher drug-resistant index.

Expressions of P-gp and MRP in the three groups of drug-resistant cells were significantly increased compared with the parental generation. The expression of GSH/GST in the three groups showed no statistical significance by paired comparison (*P *> 0.05). Our results suggested that the high expression of P-gp might induce the decrease of intracellular ADM accumulation and constitute a molecular basis of drug resistance. Moreover, MRP over-expression might be another molecular basis of drug resistance. Nevertheless, there was no significant relationship between the formation of drug resistance of hepatoma carcinoma cell and the expression of GSH/GST.

### Advantages and disadvantages of in vitro induction and in vivo induction

Our study proved that the superiority of a drug-resistant cell model established by the in vitro concentration gradient incremental method is that the drug-resistant index and stability were high. The disadvantage was that cell proliferation was quite low. The induction of the drug-resistance process wasted much time and it was easier to induce contaminants during the induction. The superiority of the drug-resistance model established by nude mice in vivo induction was due to its stronger reproductive activity, short time of induction (generally about 8 weeks) and the low possibility of contamination. However, the disadvantages mainly included the inferior drug resistance and stability.

In conclusion, we considered the drug resistance model established by the two kinds of methods based on nude mice in vivo introduction was comparatively ideal. Firstly, stable drug resistance was involved in both methods. Secondly, both methods reflected the formation of clinical drug resistance accurately. Both of the modeling methods and medications during chemotherapy were quite similar; large doses of chemotherapeutics were injected into the living body in a short time and reached a certain blood drug level to kill the cancer cells. Clinically, large doses and short-range administrations [22] are commonly used to relieve the side effect of chemotherapeutics and to improve the therapeutic effect. Similar to the clinical drug-resistant cells, all cells had quite strong reproductive activity. Patients with multi-drug resistance have recurrence or metastasis of primary tumors [23] which indicates that the drug-resistant cells appearing clinically show quite strong proliferative and metastatic ability. Tumor cell groups selected by the effects of drugs had stronger survival superiority and were able to overcome the inhibition of chemotherapy to keep normal growth and proliferation. The short time of the induction, lower possibility of contamination and the relatively simple operation are also its merits.

### Comparison of the two in vivo induction methods

We compared the two drug-resistance models with nude mice in vivo implantation progressively. Our results validated that aspects such as cell morphology, multiples of drug resistance, the influx and efflux of drug and the variation of P-gp, MRP and GSH/GST were all fundamentally similar. The advantages of subcutaneous implantation were due to its simple operation and easy observation. However, the tumor growth environment was different from human hepatoma; the tumor growth was relatively local and generated limited distant metastases. The superiority of liver implantation was quite obvious, especially in tumor growth environment, location and biological behavior were quite similar to human hepatoma, the proportion of the genesis of tumor metastasis, infiltration and ascites were quite high. Therefore, the drug-resistance model established by nude mice liver implantation was capable of better simulating human hepatoma. The ideal model has similar characteristics of human heptoma biology and the pharmacokinetics of anti-cancer drugs. The utilization of this model not only allows the exploration of the molecular mechanism of hepatoma multi-drug resistance with multiple angles and targets, but also provided an ideal experiment using plates for the screening of hepatoma drug-resistant reversal agents.

## Competing interests

The authors declare that they have no competing interests.

## Authors' contributions

XGZ carried out the molecular genetic studies, participated in the sequence alignment and drafted the manuscript. MMX participated in the sequence alignment. RHG participated in the design of the study and performed the statistical analysis. XLM conceived of the study, and participated in its design and coordination and helped to draft the manuscript. All authors read and approved the final manuscript.
